# Methylglyoxal Has Different Impacts on the Fungistatic Roles of Ammonia and Benzaldehyde, and Lactoylglutathione Lyase Is Necessary for the Resistance of *Arthrobotrys oligospora* to Soil Fungistasis

**DOI:** 10.3389/fcimb.2021.640823

**Published:** 2021-04-28

**Authors:** Xi Long, Nian-Min He, Li-Xue Tan, Yun-He Yang, Jia-Peng Zhou, Zi-Yi Liu, Ming-He Mo, Tong Liu

**Affiliations:** ^1^ State Key Laboratory for Conservation and Utilization of Bio-Resources in Yunnan, Yunnan University, Kunming, China; ^2^ Technical Center, Puer Corporation of Yunnan Tobacco Corporation, Puer, China; ^3^ Biocontrol Engineering Research Center of Crop Disease and Pest in Yunnan Province, Yunnan University, Kunming, China

**Keywords:** lactoylglutathione lyase, methylglyoxal, soil fungistasis, ammonia, benzaldehyde

## Abstract

Biocontrol of root-knot nematode has attracted increasing attention over the past two decades. The inconsistent field performance of biocontrol agents, which is caused by soil fungistasis, often restricts their commercial application. There is still a lack of research on the genes involved in biocontrol fungi response to soil fungistasis, which is important for optimizing practical applications of biocontrol fungi. In this study, the lactoylglutathione lyase-encoding *AOL_s00004g335* in the nematophagous fungi *Arthrobotrys oligospora* was knocked out, and three mutant strains were obtained. The hyphal growth of mutants on the three media was almost the same as that of the wild-type strain, but mutants had slightly higher resistance to NaCl, SDS, and H_2_O_2_. Methylglyoxal (MG) significantly increased the resistance of *A. oligospora* to ammonia, but decreased the resistance to benzaldehyde. Furthermore, the resistance of the mutants to soil fungistasis was largely weakened and MG could not increase the resistance of *A. oligospora* to soil fungistasis. Our results revealed that MG has different effects on the fungistatic roles of ammonia and benzaldehyde and that lactoylglutathione lyase is very important for *A. oligospora* to resist soil fungistasis.

## Introduction

Plant-parasitic nematodes cause much more annual damage compared to pests, and they lead to more than 100 billion dollars of global agricultural loss every year (Coyne et al., 2018). The root-knot nematode (RKN), *Meloidogyne* spp., accounts for about 50% of the losses caused by all plant-parasitic nematodes (Abad et al., 2008; Singh et al., 2015). The RKN also causes plant wounds, through which microbial pathogens infect the plant, and this frequently leads to the underestimation of the damage caused by nematodes (Basso et al., 2020). In the past decades, the main control method against *Meloidogyne* spp. has been chemical nematicides. However, many chemical nematicides (e.g., methyl bromide) have been forbidden in recent years because of their strong toxicity (Mao et al., 2017). Avermectin, fosthiazate, and fluopyram are the main chemical nematicides used at present. Except for pesticide residues in food, the prolonged use of nematicides also results in drug resistance in nematodes (Wolstenholme et al., 2004; Ghosh et al., 2012), so new alternative methods for controlling nematode diseases are necessary. Nematode biocontrol agents can meet these requirements, and has been attracting more and more attention from researchers (Li et al., 2015; Zhang et al., 2020).

Great effort has been made by scientists to develop nonchemical and eco-friendly RKN management strategies. Many microorganisms, such as *Pochonia chlamydosporia* (Atkins et al., 2003), *Purpureocillium lilacinum* (Mo et al., 2019), *Muscodor albus* (Riga, 2008), *Trichoderma harzianum* (Sharon et al., 2001), and *Arthrobotrys oligospora* (Singh et al., 2013) are effective in controlling RKN. Although some of these microbes have been developed as nematicides, inconsistent field performance often restricts the commercial development of biocontrol agents against RKN, and biocontrol microbes are often not considered as acceptable alternatives for pesticides (Chen et al., 2020). This inconsistency can be caused by a large number of biotic and abiotic factors in soil, including interaction with non-target organisms, damage by other pathogens and pests, degree of rhizosphere colonization, and physical and chemical composition of the rhizosphere (Zhang et al., 2020). The outcomes of some of these factors are named as soil fungistasis.

Dobbs and Hinson first proposed the concept of soil fungistasis in 1953 to describe the widespread occurrence of the inhibition of fungal spore germination or growth of fungal hyphae in the soils (Dobbs and Hinson, 1953). Regarding the mechanism of fungistasis generation, decades of research have indicated that the combined role of nutritional deficiency and inhibitory factors induces it (Garbeva et al., 2011). Several studies have shown that nutritional competition is related to soil fungistasis (Lockwood, 1964; Mondal and Hyakumachi, 1998; Legrand et al., 2019). In contrast, many inhibitory factors have been identified, including aluminum (Ko and Hora, 1972), ammonia (Ko, 1974), and ethylene (Balis, 1976). Since 2000, many volatile fungistatic compounds originating from soil microorganisms have been reported, including methylamine, trimethylamine, acetamide, and benzaldehyde (Xu et al., 2004; Zou et al., 2007). Production of ammonia by *Streptomyces* species was shown as a low-cost and long-distance antibiotic strategy (Avalos et al., 2019). Furthermore, there are several indications that soil microbial community structure, activity and diversity are determinants of soil fungistasis (Termorshuizen et al., 2006; Janvier et al., 2007; Rotenberg et al., 2007). Moreover, interactions within the soil microbial community may play a significant role in soil fungistasis (Wietse et al., 2007; Garbeva, 2010). After almost seven decades of research, it is clear that soil microbial activities lie at the heart of fungistasis, and these activities result in fungal nutrient deficiency or accumulation of fungistatic compounds (Garbeva et al., 2011; Li et al., 2020).

Although the causes of soil fungistasis are almost clear after decades of research, the molecular mechanisms underlying how soil fungistasis repress the germination and growth of fungi, and how fungi respond to soil fungistasis have not been elucidated. These issues are important to optimize practical applications of biocontrol fungi against RKN. Quantitative proteomics revealed proteomic changes in the conidia of the nematode-trapping fungus *Arthrobotrys oligospora* in response to two fungistatic factors, ammonia (Liu et al., 2018) and benzaldehyde (Liu et al., 2019). The functions of these differentially regulated proteins in response to ammonia or benzaldehyde are yet to be fully determined.

Among these proteins, lactoylglutathione lyase (EC:4.4.1.5), encoded by *AOL_s00004g335*, was upregulated more than two folds under fungistatic stress induced by ammonia or benzaldehyde. Lactoylglutathione lyase belongs to the glyoxalase system, which is a detoxification system of methylglyoxal (MG). MG is a by-product of glycolysis and a highly reactive substance with strong oxidant and glycosylation properties (Leoncini et al., 1989; Nomura et al., 2010; Bankapalli et al., 2015). The accumulated MG can react with proteins, DNA and other biomolecules, leading to irreversible structural damage and loss of function (Inoue and Kimura, 1995; Du et al., 2006; Shaheen et al., 2014). Therefore, the elimination of MG by the glyoxalase system is essential (Toth et al., 2014). It has been reported that MG concentration in various plant species increases 2 to 6 folds in response to salinity, drought, and cold. The accumulation of MG results in the inhibition of seed germination, and the lactoylglutathione lyase plays an important role in maintaining MG levels in plants under normal and abiotic stress conditions (Yadav et al., 2005). In this study, we aimed to reveal the function of MG and lactoylglutathione lyase in soil fungistasis.

## Materials and Methods

### Strains and Vectors

The nematode-trapping fungus *Arthrobotrys oligospora* ATCC24927 was purchased from the American Type Culture Collection and maintained on corn meal agar (CMA) plate at 4°C (Yang et al., 2013). Its genome, containing a 40.07-Mb assembled sequence, was reported by our lab in 2011 (Yang et al., 2011). Plasmid pCSN44 was stored in *Escherichia coli* strain DH5а, and used to amplify the hygromycin B resistance gene *hph*, which is a selection marker for a gene knockout (Jiang et al., 2017; Zhang et al., 2019). Plasmid pRS426 was used as a backbone plasmid for constructing a gene knockout plasmid, and *Saccharomyces cerevisiae* FY834, a uracil auxotrophic strain, was used as a host strain for recombinational cloning procedures (Xie et al., 2019; Xie et al., 2020).

### *AOL_s00004g335* Knockout Vector Construction

The disruption vector of *AOL_s00004g335* was constructed using a modified yeast cloning procedure (Li et al., 2019; Wang et al., 2019). The 2183 bp upstream homologous fragment and the 1956-bp downstream homologous fragment of *AOL_s00004g335* were amplified *via* PCR using primer pairs 335-5f/5r, and 335-3f/3r, respectively (**Supplementary Materials, Table S1**). The hygromycin cassette was obtained *via* PCR using plasmid pCSN44 as a template, and the primer pair hphF/hphR was also used. The amplified fragments had 21-bp tails homologous to the hygromycin cassette (*hph*). The two homologous fragments of *AOL_s00004g335* were co-transformed into yeast strain FY834 along with the *hph* cassette and gapped yeast shuttle vector (pRS426). The endogenous homologous recombination system of yeast created a circular plasmid, and the final disruption vector (pRS426-g335-hph) was recovered *via* transformation into *E. coli*. The recombinant plasmid extracted from *E*. *coli* DH5a was confirmed as the correct plasmid using PCR and DNA sequencing.

### Protoplast-Based Gene Knockout

The *A*. *oligospora* ATCC24927 was cultured in 250 ml Erlenmeyer flask containing 150 ml liquid TG (1 % tryptone, and 1 % glucose) medium for 36 h at 28°C, and then the hyphae were collected and used to prepare protoplasts according to a previously described method (Tunlid et al., 1999; Leng et al., 2008; Park et al., 2011). The knockout cassette fragment was amplified with primers 335-5f and 335-3r using plasmid pRS426-g335-hph as a template, and then transformed into *A. oligospora* following a protoplast-based protocol (Tunlid et al., 1999; Leng et al., 2008). Next, the transformants were selected and confirmed *via* PCR according to a previously reported method (Liu et al., 2018; Liu et al., 2019), using primers yz-5f and yz-3r (**Supplementary Table S1**).

### Hyphae Growth and Conidia Yield of the Wild-Type and Mutant Strains Under Different Conditions

The hyphae growth of wild-type and mutant strains was measured on 9 cm plates containing PDA, TG, and TYGA solid media at 28°C according to a previously reported method (Li et al., 2019). The radius of colonies was measured every day for 5 days.

To compare the stress response capabilities, the hyphae growth of these strains was also tested on 9 cm plates using TG medium which contained the following: 0.01% and 0.02% SDS; 0.1, 0.2, and 0.3 M NaCl; 5, 10, and 15 mM H_2_O_2_; 1, 2, and 4μl MG solution (~40% in water, Sigma-Aldrich, USA) per 5 mL TG medium. The hyphal growth was evaluated by measuring the radius of the colonies for 5-7 days.

The sporulation capacity of these strains was analyzed according to the following method. Hypha plugs (0.5 cm in diameter) from the wild-type and mutant strains were inoculated at the center of Corn Meat Agar medium in 250 mL Erlenmeyer flasks and cultured at 28°C for 14 days. Then, 30 ml of sterile water and properly sterilized glass beads were added into the Erlenmeyer flask and shaken slightly. After that, 30 ml water-containing conidia was filtered to remove the hyphae debris using six layers of lens paper, and 10 μl of conidia suspension was used to count the number of conidia using a hemocytometer.

Three replicates of all the experiments described above were performed.

### Comparison of Conidial Germination Rates Between the Wild-Type and Mutant Strains Under Normal or Methylglyoxal Suppression Conditions

The conidia of the wild-type and mutant strains were collected according to the method mentioned above. The conidia suspension was then cultured in 100 mL Erlenmeyer flasks containing deionized water, placed in constant temperature shaker, and shook at 160 rpm at 28°C. Spore germination rates were observed microscopically after 0, 4, 8, and 12 h according to described method (Liu et al., 2018).

To test the conidial germination rate under methylglyoxal (MG) suppression, 100 μl conidia suspension of wild-type or mutant strains were spread on 2% water agar (WA) plates supplemented with MG. Precisely 5 ml of WA medium, containing 2, 3, 4, 5, and 6 μl of MG solution (~40%, Sigma-Aldrich^®^), was prepared. The plates were sealed with two layers of parafilm (Solarbio, USA), and cultured at 28°C for 24 h. Then, conidial germination rates were detected.

Three replicates of all the experiments described above were performed.

### Mutant Strain Resistance to Fungistatic Stress of Ammonia or Benzaldehyde

Fungistasis was often quantified based on spore germination *via* microscopic observation (Lockwood, 1977), and the ability of mutant strains to resist the fungistatic stress of ammonia or benzaldehyde was quantified by detecting the conidial germination rates. Precisely 5 ml of WA medium was added to one side of a two-compartment Petri dish (9cm in diameter), and 100 μl of conidial suspension was spread on the WA medium. Exactly 6, 6.5, 7, 8, and 9 μl of ammonia water (25-28%, Guanghua Sci-Tech Co., Ltd., China) or 0.4, 0.6, 0.8, and 1.0 μl of benzaldehyde, respectively, was dropped on the sterilized cotton on the other side of the Petri dish. After sealing with parafilm, the plates were cultured in incubator (Tiancheng experimental instrument, Shanghai, China) at 20°C for 24 h, and then conidial germination rates were detected. Three replicates were performed.

### Evaluating the Effect of MG on the Fungistatic Role of Ammonia or Benzaldehyde

To evaluate the effect of MG on the fungistatic role of ammonia or benzaldehyde, 5 mL of WA medium, containing different volumes of MG solution, was added to one side of a two-compartment Petri dish. Three replicates were set. Other procedures were the same as the method mentioned above. The conidial germination rate of wild-type and mutant strains was detected after 24 h.

### Wild-type and Mutant Strain Resistance to Soil Fungistasis

According to the reported method (Liu et al., 2020), soil and deionized water were added to a beaker at a mass ratio of 2.5:1, 1:1, and 1:2.5, and were mixed well to produce a high fungistatic soil suspension, a medium fungistatic soil suspension, and a low fungistatic soil suspension, respectively. The fresh conidia of wild-type and mutant strains were transferred into a dialysis bag (300 kDa; Spectrum, USA), and the dialysis bag was then placed into three kinds of soil suspension at 28°C, with the agitation of the soil suspension using a magnetic stirrer, as described previously (Liu et al., 2020). After 24 h, the dialysis bags were removed from the soil suspension, and the conidia germination rate was tested microscopically.

In addition, to test the ability of exogenous MG in inducing *A. oligospora* resistance to soil fungistasis, 1 ml MG was added to 3 ml of conidia suspension, and incubated at 28°C for 5, 10, 15, 20, 25, and 30 min. Then, MG was removed by centrifugation, and the conidia were collected and resuspended in 3 ml sterilized water. Finally, the conidia suspension was transferred into a dialysis bag, placed into a soil suspension, and used to detect the conidia germination rate as described above.

Three replicates of all the experiments were performed in this section.

### Statistical Analyses

All the statistical analyses were performed using GraphPad Prism 8.0.1 (GraphPad Software Inc., San Diego, CA, USA). Multiple t test of this software was used to analyze the germination rates among different samples.

## Results

### Verification of Gene Knockout Mutants

**Figure 1:**
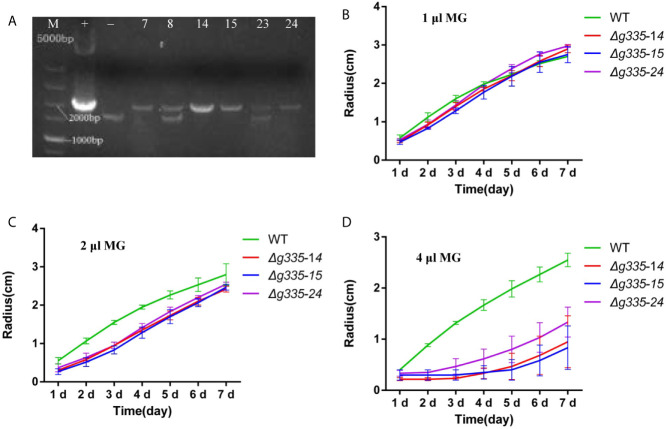
Verification of *AOL_s00004g335* knock-out mutants. **(A)** PCR verification of knock-out mutants. +, positive control. -, negative control. M, DNA marker. No. 14, 15, and 24 are positive transformants. **(B–D)** Verification of the knock-out mutants by measuring the hyphal growth on the TG medium containing different amounts of MG. 1, 2, 4 μl of MG solutions per 5 ml medium were used in **(B–D)**, respectively. WT, wild-type strain. *Δg335-14*, *Δg335-15*, and *Δg335-24*: *AOL_s00004g335* knock-out mutants.

To study the function of lactoylglutathione lyase and MG in response to fungistatic stress, the *AOL_s00004g335*, encoding lactoylglutathione lyase, was knocked out. The genomic DNA of hygromycin-resistant transformants was isolated and used as templates for PCR verification of knockout mutants, using the genomic DNA of the wild-type strain as a negative control and the plasmid pRS426-g335-hph as a positive control. The transformant, which had a 2018-bp amplified fragment similar to the positive control, was positive, and the transformants with 1638-bp and 2018-bp amplified fragments were negative. Three positive transformants (g335-14, g335-15, and g335-24) were obtained (**Figure 1A**). Further verification of the knockout mutants was performed by measuring their hyphal growth on the TG medium containing different amounts of MG. On TG medium containing 1 μl MG solution per 5 ml medium, the hyphal growth of three transformants (*Δg335-14, Δg335-15*, and *Δg335-24*) were almost the same as that of the wild-type strain (**Figure 1B**). However, when MG concentration in the TG medium increased, the hyphal growth of the three transformants was severely impaired (**Figures 1C, D**). This result suggested that the resistance of mutants to MG was reduced and indirectly verified that the strains *Δg335-14, Δg335-15*, and *Δg335-24* were positive transformants.

### The Effect of *AOL_s00004g335* Deletion on Hyphal Growth and Stress Resistance

**Figure 2:**
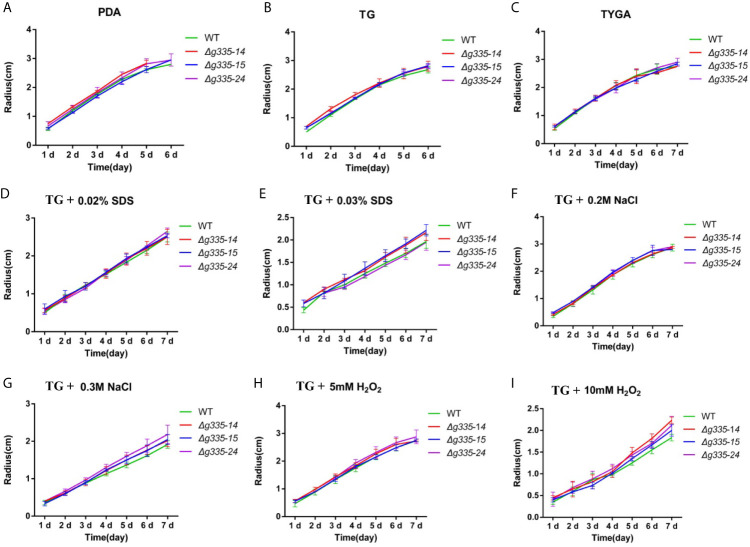
Measurement of the hyphal growth of mutants under normal or stressful condition. **(A–C)** The hyphal growth rates of mutant and wild-type strain on PDA, TG, and TYGA media. **(D, E)** The hyphal growth rates of mutant and wild-type strain on TG medium containing 0.02% **(D)**, and 0.03% **(E)** sodium dodecyl sulfate (SDS). **(F, G)** The hyphal growth rates of mutant and wild-type strain on TG medium containing 0.2M **(F)**, and 0.3M **(G)** sodium chloride (NaCl). **(H, I)** The hyphal growth rates of mutant and wild-type strain on TG medium containing 5mM **(H)**, and 10 mM (Fig. I) hydrogen peroxide (H_2_O_2_). WT, wild-type strain. *Δg335-14*, *Δg335-15*, and *Δg335-24*: *AOL_s00004g335* knock-out mutants.

To examine whether the disruption of lactoylglutathione lyase influenced the growth of *A. oligospora*, the hyphal growth of wild-type and mutant strains on PDA, TG, and TYGA media was determined (**Figures 2A–C**). Compared with the wild-type strain, the three mutant strains had almost the same growth rate, indicating that *AOL_s00004g335* deletion had no obvious influence on hyphal growth. In addition, there was no notable difference in the sporulation capacity between these mutants and the wild-type strain (data not shown).

The growth rates of wild-type and mutant strains were also compared on the TG medium with different stress factors, including NaCl, SDS, and H_2_O_2_. The mutant strains exhibited the same growth rate as wild-type on the TG medium containing a low concentrations of NaCl (0.2 M), SDS (0.02%), and H_2_O_2_ (5 mM) (**Figures 2D, F, H**), but the growth rates of the mutant strains were slightly faster than those of the wild-type on the TG medium containing high concentrations of NaCl (0.3 M), SDS (0.03%), and H_2_O_2_ (10 mM) (**Figures 2E, G, I**). These results indicated that the mutant strains had relatively higher stress resistance to some extent.

### *AOL_s00004g335* Deletion Increases Ammonia Resistance of Mutant Strains Significantly

**Figure 3:**
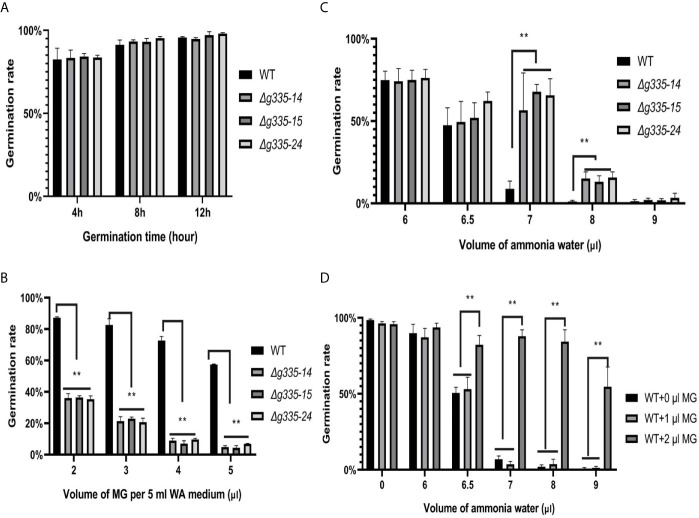
Measurement of ammonia resistance of mutant strains on WA medium. **(A)** Determination of conidial germination rates of mutant and wild-type strains at 4, 8, and 12 hours. **(B)** Determination of conidial germination rates (24 h) of mutant and wild-type strains on WA medium containing different volume of MG. **(C)** Determination of the fungistatic effect of ammonia on the conidial germination rates (24h) of mutant and wild-type strain. **(D)** Exogenous MG relieved the fungistatic inhibition of ammonia on the conidial germination of wild-type strain. WT, wild-type strain. *Δg335-14*, *Δg335-15*, and *Δg335-24*: *AOL_s00004g335* knock-out mutants. **P < 0.01.

Compared with the wild-type strain, the conidial germination rates of *A. oligospora* on WA medium after 4, 8, and 12 h were not influenced by the deletion of *AOL_s00004g335* (**Figure 3A**). However, the germination rates of mutant strains at 24 h on the WA medium containing MG decreased severely (less than 40%) even when only 2 μl 40% MG solution was added. Under the same condition, the germination rate of wild-type strain was more than 80% (**Figure 3B**). These results suggested that the lactoylglutathione lyase encoded by the *AOL_s00004g335* was very important for *A. oligospora* to detoxify MG.

The germination rates of mutant and wild-type strains were also determined and compared under the fungistatic stress of ammonia. As shown in **Figure 3C**, the ammonia resistance of the mutant strains increased significantly (p<0.01). The germination rate of the wild-type strain was less than 10% under the fungistatic stress of 7 μl ammonia water, which was far less than those of the mutant strains (56-67%). Even under the fungistatic stress of 8 μl ammonia water, there was still ~15% of conidia of mutant strains that could germinate. The deletion of *AOL_s00004g335* might result in the accumulation of MG, and we wondered whether the accumulated MG improved ammonia resistance of the mutant strains. Therefore, different amounts of 40% MG solution were added into the WA medium, and germination rates of the wild-type strain were determined under the fungistatic stress of ammonia (**Figure 3D**). Surely, 2 μl MG solution significantly improved the germination rates of *A. oligospora* under the fungistatic stress of 6.5, 7, 8, and 9 μl ammonia water.

### *AOL_s00004g335* Deletion Weakens the Benzaldehyde Resistance of Mutant Strains

**Figure 4:**
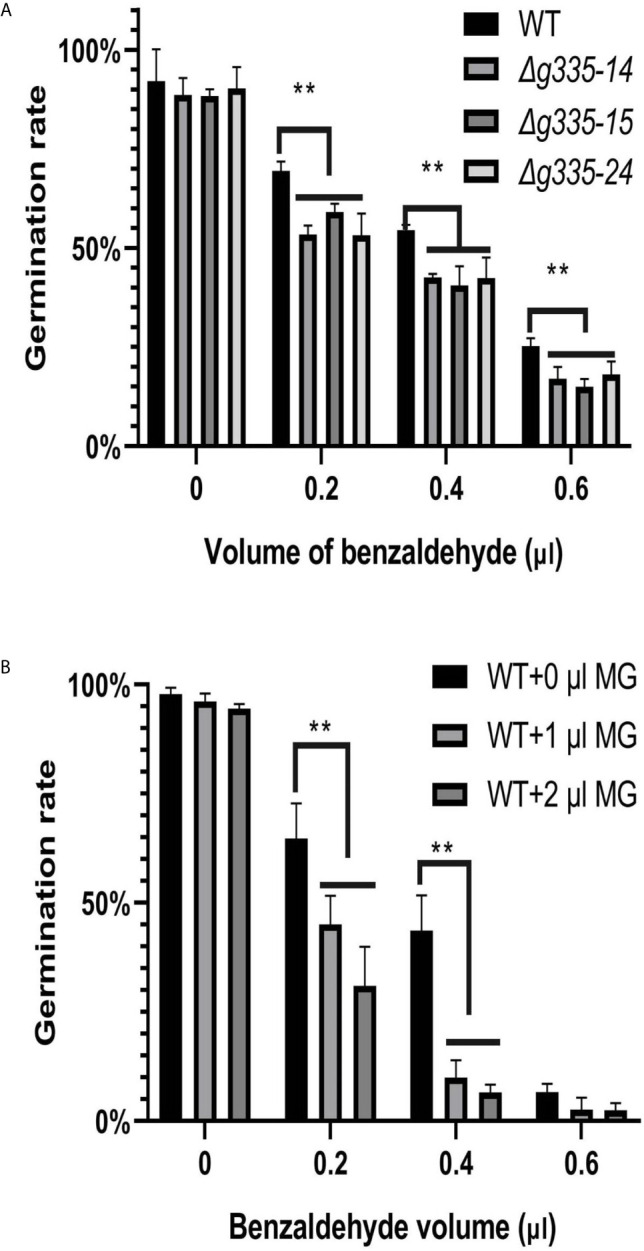
Measurement of benzaldehyde resistance of mutant strains on WA medium. **(A)** Determination of conidial germination rates (24 h) of mutant and wild-type strains on WA medium containing different volume of benzaldehyde. **(B)** Exogenous MG increased the fungistatic inhibition of benzaldehyde on conidial germination of wild-type strain. WT: wild-type strain. *Δg335-14*, *Δg335-15*, and *Δg335-24*: *AOL_s00004g335* knock-out mutants. **P < 0.01.

The above results showed that deletion of *AOL_s00004g335* increased the resistance of mutant strains against SDS, NaCl, H_2_O_2_, and the fungistatic factor ammonia. In order to evaluate the resistance of mutant strains against another fungistatic factor, we tested the benzaldehyde resistance of mutant strains by observing the conidial germination rate. As shown in **Figure 4A**, conidial germination rates of *AOL_s00004g335* knockout mutant strains were significantly (p <0.01) lower than those of the wild-type strain under the fungistatic stress of 0.2, 0.4, and 0.6 μl benzaldehyde. Besides, 1 or 2 μl of MG had almost no inhibitory effect on the conidial germination of the wild-type strain when benzaldehyde was absent, but it increased the inhibition effect of benzaldehyde (**Figure 4B**). Approximately 60% and 40% of wild-type conidia germinated in the presence of 0.2 and 0.4μl benzaldehyde, respectively; furthermore, the addition of 1 or 2 μl of MG resulted in significantly (p <0.01) lower conidial germination rates.

### *AOL_s00004g335* Is Important for *A. oligospora* Resistance to Soil Fungistasis

**Figure 5:**
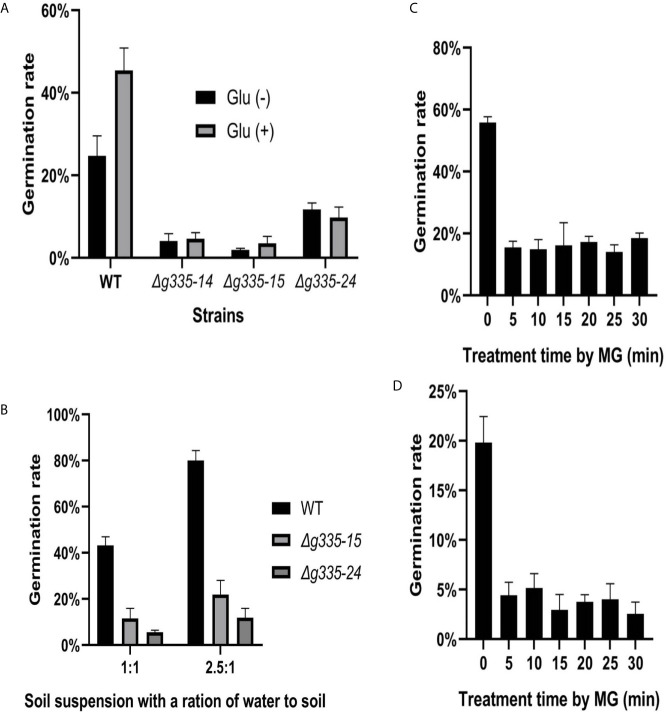
The function determination of *AOL_s00004g335* in resisting soil fungistasis. **(A)** The conidial germination rates (24 h) of mutant and wild type strains were determined in high fungistatic soil suspension (glu-), and the relief effect of conidial germination rates by glucose was compared in high fungistatic soil suspension (glu+). **(B)** Determine the conidial germination rates (24h) of mutant and wild type strains in medium fungistatic soil suspension (1:1), or low fungistatic soil suspension (2.5:1). **(C, D)** The conidia of wild type strain were treated by MG for different time, then placed in high fungistatic soil suspension **(C)**, or medium fungistatic soil suspension **(D)** for 24h, and determine the conidial germination rate. WT, wild-type strain. *Δg335-14*, *Δg335-15*, and *Δg335-24*: *AOL_s00004g335* knock-out mutants. Glu(-), conidia suspension without glucose. Glu(+), conidia suspension with glucose.

The deletion of *AOL_s00004g335* resulted in different responses of *A. oligospora* to fungistatic factors ammonia and benzaldehyde. The function of this gene in response to soil fungistasis was further studied. Compared to that of the wild-type strain, the conidial germination rate of the three mutant strains (*Δg335-14, Δg335-15*, and *Δg335-24*) decreased significantly in the high fungistatic soil suspension (**Figure 5A**), and the relief effect of soil fungistasis by glucose observed in the wild-type strain disappeared in the mutant strains. In addition, the conidial germination of mutant strains was worse than that of the wild-type strain in soil suspensions with less fungistatic intensity (**Figure 5B**), especially in low fungistatic soil suspension. This soil suspension had a little fungistatic effect on wild-type conidia, which had a germination rate of ~80%, but the mutant strains had a germination rate of less than 20%. These results suggested that lactoylglutathione lyase encoded by *AOL_s00004g335* is important for *A. oligospora* resistance to soil fungistasis.

We wondered whether exogenous MG can induce the resistance of *A. oligospora* to soil fungistasis. Hence, the conidia of *A. oligospora* were treated with approximately 2.17 μM MG for 5 to 30 min, and the conidial germination rates were detected in high, and medium fungistatic soil suspensions (**Figures 5C, D** **)**. The results showed that none of the treatments improved the resistance of *A. oligospora* to soil fungistasis; conversely, all treatments significantly reduced conidial germination rates (p <0.01).

## Discussion

Here, we aimed to reveal the function of MG and lactoylglutathione lyase in soil fungistasis and found that that MG had contrary effects on the fungistatic roles of ammonia and benzaldehyde. MG relieved the fungistatic role of ammonia, but increased the fungistatic role of benzaldehyde. Moreover, lactoylglutathione lyase is necessary for *A. oligospora* to resist soil fungistasis.

Resistance to soil fungistasis is very important for RKN biocontrol fungi to exert their control effect; however, there is little known about this. Based on proteomics data, it was found that lactoylglutathione lyase was upregulated more than two folds under fungistatic stress induced by ammonia or benzaldehyde. Lactoylglutathione lyase belongs to the glyoxalase system and detoxifies MG. Our results showed that lactoylglutathione lyase was the main detoxification enzyme of MG, and its deletion had no negative effect on the growth of *A. oligospora* on PDA, TG, and TYGA; furthermore, the mutant strains had even higher resistance to NaCl, SDS, H_2_O_2_ (**Figure 2**), and ammonia (**Figure 3C**). This might be due to the signaling function of MG that might accumulate in mutant strains after the deletion of lactoylglutathione lyase. It was reported that MG concentration varies between 30–75 μM in various plant species and it increases 2 to 6 folds in response to salinity, drought, and cold stress conditions (Yadav et al., 2005), and it was considered as a signal molecule, which can improve the resistance of plants to abiotic stress by activating the glyoxalase system and oxidation-antioxidation system (Bless et al., 2017; Li et al., 2017a; Li et al., 2017b; Majláth et al., 2020). Moreover, our results were consistent with the results of these studies.

Surprisingly, exogenous MG significantly increased the resistance of *A. oligospora* to fungistasis by ammonia. No report suggests that MG can react with ammonia directly, but MG may well be transformed into other compounds *in vivo*, mainly lactate (Ghosh et al., 2016; Bari et al., 2019), which can react with ammonia. In addition, the induced resistance of *A. oligospora* to ammonia by exogenous MG might exist because 1 μl of MG hardly increased the resistance of *A. oligospora* to ammonia (**Figure 3D**). However, the resistance supplied by MG was not always effective; for example, MG increased the fungistatic role of benzaldehyde, and there was a synergistic effect between benzaldehyde and MG (**Figure 4**).

The fungistatic role of benzaldehyde can partially attribute to the cellular toxicity of MG. In our recent research on *A. oligospora* ATCC24927, we found that lactoylglutathione lyase was upregulated more than two folds under the fungistatic stress induced by ammonia or benzaldehyde (Liu et al., 2018; Liu et al., 2019). Although the obvious accumulation of MG in conidia was not detected using high-performance liquid chromatography under these two fungistatic stresses (Data not shown), the upregulation of lactoylglutathione lyase still suggested the accumulation of MG (Zuin et al., 2005). On the one hand, 2 μl of MG (about 2.6 μM) inhibited the conidial germination of mutant strains significantly (**Figure 3B**), it means the concentration of MG in conidia is far lower than that in the plant. And on the other hand, MG is easy to associate with proteins and DNA (Inoue and Kimura, 1995; Shaheen et al., 2014; Toth et al., 2014); therefore, most of the produced MG in conidia may not be dissociated and detected.

Although the proteomics data shown that lactoylglutathione lyase was upregulated more than two folds under fungistatic stress induced by ammonia or benzaldehyde, it is difficult to evaluate enzyme activity of lactoylglutathione lyase in conidia. There are four MG degradation pathways in eucaryotic cell (Mostofa et al., 2018), lactate is the degradation product in three of the pathways, including the lactoylglutathione lyase pathway. And lactate can also be produced by glycolytic pathway. what’s more, MG is a high reactive molecule which can react with proteins and DNA. So, it difficult to evaluate enzyme activity by measuring the consumption of MG or production of lactate. Besides, after the deletion of *AOL_s00004g335*, compensatory degradation of MG by other pathways would increase. Under fungistatic stress induced by ammonia or benzaldehyde, upregulation of lactoylglutathione lyase could help conidia limit MG to low concentration and avoid the toxicity of high concentration MG.

This study also suggested that MG might be involved in the fungistatic role of soil, and the detoxification of MG is important for *A. oligospora* to resist soil fungistasis. Compared to benzaldehyde, the lactoylglutathione lyase played more important role in soil fungistasis. Although MG can also activate the stress response in fungi (Zuin et al., 2005; Takatsume et al., 2006), compared to a single fungistatic factor, soil is a very complex fungistatic environment containing many fungistatic factors, the stress response induced by exogenous MG (2.17μM) was not enough for *A. oligospora* to resist soil fungistasis; in contrast, it enhanced the fungistatic role of soil (**Figures 5C, D** **)**.

This study provides an insight into the participation of MG in the fungistatic role of several factors, including soil, and enriched our understanding of stress response to soil fungistasis in the nematode-trapping fungi.

## Data Availability Statement

The original contributions presented in the study are included in the article/**Supplementary Material**. Further inquiries can be directed to the corresponding authors.

## Author ContributionS

Authors XL, N-MH, and L-XT contributed equally to this work. XL and N-MH carried out the main experiments. L-XT mainly drafted the manuscript. M-HM and TL designed the study, analyzed the data, and revised the manuscript. All authors contributed to the article and approved the submitted version.

## Funding

This research was financed by the National Natural Science Foundation Program of China (31960022 and 31870091), the Department of Science and Technology of Yunnan Province (202001BB050057 and 2019ZG00901), and the Ten-thousands Talents Program in Yunnan Province (YNWR-CYJS-2019042 and YNWR-QNBJ-2018153). The authors declare that this research was also financed by China Tobacco Yunnan Industrial Co. Ltd. (2019530000241018). The funder had no involvement with the study.

## Conflict of Interest

Author Z-YL was employed by Puer Corporation of Yunnan Tobacco Corporation.

The remaining authors declare that the research was conducted in the absence of any commercial or financial relationships that could be construed as a potential conflict of interest.
