# Supplementary material for: Methylglyoxal Has Different Impacts on the Fungistatic Roles of Ammonia and Benzaldehyde, and Lactoylglutathione Lyase Is Necessary for the Resistance of Arthrobotrys oligospora to Soil Fungistasis
Source: Front Cell Infect Microbiol. 2021 Apr 28;11:640823. doi: 10.3389/fcimb.2021.640823 (PMC8113876; doi:10.3389/fcimb.2021.640823)
Supplement: Supplementary file 1 [file Table_1.doc]

| Primer name | Sequence (5’-3’) |
| --- | --- |
| 335-5f | GTAACGCCAGGGTTTTCCCAGTCACGACGCATTTGTCCTATTAGCCCCC |
| 335-5r | ATCCACTTAACGTTACTGAAATCTCCAACCCTTTTCTTCCGAGTTTTCTTT |
| 335-3f | CTCCTTCAATATCATCTTCTGTCTCCGACAGGTTGACTGATGGCAAGATG |
| 335-3r | GCGGATAACAATTTCACACAGGAAACAGCAACGAGCAAGACCACACTAAAA |
| hphF  hphR  yz-5f | GTCGGAGACAGAAGATGATATTGAAGGAGC  GTTGGAGATTTCAGTAACGTTAAGTGGAT  TAATGCTCATACCCCGAATC |
| yz-3r | GTTTACCAGTCTGCTCGCTT |

Table S1 Primers used in this study
